# Elongation of the Cervix Prolapsus and Procidenture Vaginae Et Uteri Principles and Operative Treatment

**Published:** 1905-06

**Authors:** A. Belcham Keyes

**Affiliations:** Professor of Gynecology Chicago Policlinic, Instructor Gynecology and Obstetrics Rush Medical College, in affiliation with the University of Chicago; attending Gynecologist to Policlinic Chicago Maternity and Deaconess’ Hospitals, etc.


### ELONGATION OF THE CERVIX PROLAPSUS AND PROCIDENTURE VAGINAE ET UTERI PRINCIPLES AND OPERATIVE TREATMENT.

BY A. BELCHAM KEYES, M.D.

Professor of Gynecology Chicago Policlinic, Instructor Gynecology and Obstetrics Rush Medical College, in affiliation with the University of Chicago; attending Gynecologist to Policlinic Chicago Maternity and Deaconess' Hospitals, etc. *Lecture delivered to the Post-Graduate Students at the Chicago Policlinic, February, 1905.*

The subject of this lecture is one of more than passing importance, and it has often occurred to me as too little considered in its true gravity by the profession at large, and that many of the cases of prolapse are due to a lack of specific prophylaxis during labor and the puerperium.

(a) *The Elongation of the Cervix* should be considered first because it has been so frequently taken for a prolapse of the uterus, and has much the same appearance at first sight, and indeed, in some cases both elongation of the cervix and prolapsus uteri occur together.

The elongation (especially in its supra-vaginal portion) of the cervix is from much the same cause as prolapsus uteri, viz: prolapse of the vagina, which so drags at its cervical (uterine) attachment, and *failing to drag the uterus down, causes the cervix to elongate as a result.* The corpus and fundus uteri still remain at their normal level (or nearly so), being either retained by adhesions or a tumor, or other cause; or if the position of the uterus be one of normal physiological anteversion and held so by strong, healthy elastic uterine ligaments (*round, broad, utero-sacral and utero-vesical*) which consequent on their contractile action *retain the uterus at right angles to the vagina*, so that the uterus does not prolapse, even though there are present certain predisposing factors, e. g. tear or relaxation of the perineum, unless the patient exposes herself to a very great increase of abdominal pressure, e. g., carrying heavy weights, coughing, sneezing, straining at stool, etc.

(b) *Vaginal Prolapse*, which in reality is usually the prime aetiologic factor in both (1) cervix elongation and (2) prolapsus uteri, deserves our special consideration of both the physiologic and pathologic predisposing changes it undergoes, viz:

(1). *Nulliparae* often show a markedly loose condition of the para-metric and para-vaginal tissues, and a great tendency to retroversion and primary prolapsus uteri, due probably to a congenital or acquired deficiency in the elasticity in the broad and other ligaments, especially if subjected suddenly to very great increase in abdominal pressure.

(2). *In pregnancy* the vagina both widens and lengthens, with increased loosening and mobility of its surrounding *para-vaginal* connective tissue.

(3). *In labor* the child, if large, causes a marked dilitation, especially marked in rapid forceps-delivery or extraction, or in case of the premature use of ergot, the latter practice being (I have learned in my clinic) more common among midwives to-day than is generally believed.

(4). *In the puerperium* the involutic of the vaginal tissue is never entirely to its former narrow nulliparous dimensions, the increased length and width of pregnancy and labor remain in part, as well as much para-vaginal looseness, and often perineal tears.

The *perineum* which anatomically extends from the clitoris to the anus, must now be considered, and indeed it is very important gynecologically throughout its whole extent even if the center of interest is in the so-called *central perineal tenden or body* usually styled " the *perineum.*"

The points to consider are the muscles, viz: the bulbous cavernosi, tranverse perinei, sphincter ani ext, and the (all important) levator ani, (with its recto-vesical fascia above and levator ani-fascia below) all of which with the pelvic organs they support, intermittently rise and fall with respiration, indeed this is the *Inferior Abdominal wall or floor of the abdominal cavity.*

Each of the muscles has an important office in forming a point of attachment for its opposite muscle and in maintaining the canals and openings in the pelvic floor. (a) In a certain direction in regard to the pelvic axis, and (b) at a certain angle to the normally situated organ above to which it is attached, so that *the distribution of intra-abdominal pressure is spread evenly* over the entire pelvic floor.

*The normal physiological anteverted uterus* lies a little less than, or at a right angle to the H-shaped vagina, i. e. the vagina is almost horizontal when the woman is standing; also the anus is at right angles to the rectum, and the urethra at right angles to the long axis of the bladder.

(5) *The tearing of this terineal body,* i. e. any of its component muscles, interferes materially with the direction of the vagina. If the tear is sufficiently deep and large to tear e. g. *the entire bulbus cavernosus attachment* and allow the unopposed ext. sphincter ani to pull the introitus vaginal further backwards towards the Coccyx than normal and thereby *change the vaginal direction* and render its angle to both the uterus and pelvis axis less acute and at the same time drag backwards the anus, so that the *recto-anal ampulla angle is much more acute* thereby making the ampulla recti more difficult to empty, and demanding greater contraction of the *levator ani muscles, which are also usually more or less weakened, stretched or torn at the central union of the two muscles posterior to the vagina,* and frequently together with the recto-vesical and levator ani fasciae sufficienly torn so that the unsupported ampulla recti soon bulges the posterior vaginal wall through the enlarged vaginal levator opening and gives rise to *rectocele.*

The posterior wall prolapses (except when the perineum much relaxed or torn) comparatively the less easily of the two on account of the firmer attachment to the rectum, while the anterior vaginal wall prolapses comparatively the more easily of the two due to its loose attachment by the anterior paravaginal tissues to the bladder, which latter easily prolapses as a *cystocele,* when the vaginal anterior wall support is weakened. As we can more readily see the rectocele than the cystocele we often give little heed of the latter.

It has often appeared to me that the works on obstetrics in advising the guarding of the perineum should be more explicit, and advise especial guarding also of the levator ani muscles by increased care and slowness during the passage of the head through its vaginal opening as well as over the much elongated perineum, especially during forceps delivery, which is usually performed all too quickly.

The transverse perinei muscles also if unopposed one to the other allow a wide gaping of the vulva.

(c) *Prolapsus et procidenture uteri :*

This is very distressing and usually considered as a condition per se, but is rathei as I have pointed out dependent on the failure of the four factors, viz.:

(a) The position of the uterus in normal enteversion.

(b) The condition of elasticity of the (so-called) uterine ligaments.

(c) The direction of the vagina.

(d) The condition of the pelvic floor.

*The normal position of the non-pregnant uterus,* being one of physiological enterversion or better slight anteflexion, care should be always taken to aid it to a return to this by all the means possible, viz.: care during parturition as well as during the puerperium, the great need of which will be apparent, when we consider the following points:

(a) *Tears of the cervix should* be avoided as much as possible by allowing the entire cervical dilatation to take place before the application of forceps or attempted extraction, both of which are frequently attempted too early (often due to the prompting of the friends to assist the suffering woman), and the consequent premature operation, not allowing the natural rotation and presentation of the best head diameters with resultant tears frequently extending into the paracervical tissue, i. e., the pare-metrium and the entrance of infection via this atrium.

(b) *The second point of extreme importance is extreme surgical cleanliness* both during the labor and during the puerperium.

If a slight infection after the labor, need not necessarily be grave, quo-ad-vitem. I yet feel that the obstetric conscientiousness has to be sufficiently innate to combat the idea which prevails that a little fever temperature of 100° Fahr. is not necessarily puerperal fever, often styled *"milk fever,"* because the patient recovers and that too little record is being kept of the sequelæ so long as the recovery is apparently good, yet *the sequelæ are often enough to destroy the usefulness of the woman as a member of society and cause her nulliparous sister to point her out as an example of the dire results of dreaded child-birth.*

This low grade puerperal fever has so frequently two landmarks, (1) the remaining chronic endometritis, and (2) a parametritis, which later often subsides, but not before the so-called uterine ligaments are perhaps crippled by the infiltration and later induration by increased connective tissue and consequent pressure atrophy of the smooth muscle fibers (derived from the external longitudinal layer of the uterus) whose contractile power is needed in order that the uterus be reposited in normal anteversion after retroversion by the full bladder as well as the correction of the cervix anteposition by a distended rectum.

(These same ligaments during the puerperium undergo involution only to a degree, and are always much looser than in the nulliparous, but if healthy they normally still retain their rectifying influence on the uterus, i. e., if their contractility be unimpaired.)

If the perametrium is converted into a mass of scar tissue then it is but little resistant to the constant strain and permanent retroversion may result, and if this reverted uterus does not become adherent it is now in the best possible position to descend the vagina even though the vagina be retained in (a) normal position and the perineum be intact, i. e., we have the condition for (a) *primary prolapsus uteri* in contradistinction to that in which the favoring factors lie in the primary prolapsus of the vagina dragging down a uterus despite good ligaments, i. e., the so-called (b) *secondary prolapsus uteri*, though I would rather say both factors usually are causative in the majority of cases, i. e., (c) *combined primary and secondary prolapsus*, yet some authors state positively that prolapsus uteri is most usually a secondary condition, i. e., the uterus is forcibly prolapsed by the prolapsing vaginal wall literally dragging it down till it gets well started, when the abdominal pressure will assist in its completion.

*Sub-involutio uteri* for which so much was claimed is a misnomer, and the remaining enlargement is really due to endometritic and matritic infiltration from an infection during labor or the puerperium.

The increased weight of the uterus (so-called sub-involuted uterus) certainly is a factor in prolapse because it favors permanent puerperal retroversion and retroversion prolapsus by being an especially favorable position for the *abdominal pressure* to cause the uterus to descend the vagina.

*Thirdly.* Retroversion often remains after labor, and if extremely common from the reasons given above; it is especially so when the woman lies always on her back allowing gravity to assist, yet must not forget that the anteverted uterus prolapses, indeed, it sometimes prolapses in an anteverted position. The diagnosis of prolapsus needs no remarks, the condition being so simple to discern.

(d) *The treatment of prolapsus uteri* has varied. The Euryphon are said to have suspended their patients by the feet. Smoking and allowing mice and lizards to run over the patient to frighten the uterus back into the pelvis, but it was not until Soranus's time that we read of tampons(wool pessary) being used. While we are naturally much more conversant with the pathology and mechanism of decensus uteri, yet I feel we are still sadly deficient in treatment.

(a) The first I should name would be, above all, *prophylaxis* by extreme surgical asepsis and antisepsis in labor, and during the puerperium, for in this latter the lacerated cervix often lies but just inside the introitus vagina, and indeed, on coughing or sneezing may protrude from it and come in contact with the labia, which are extremely difficult to render aseptic, or indeed, the napkin, if not antiseptic, is a probably frequent source of infection.

(b) *Tears of the cervix* (unless there is a very marked hemorrhage) should not be sutured, the possibility of carrying infection into the uterus is too great, despite its advocacy by some modern obstetricians.

(c) *Perineal lacerations* should be closed by the insertion of the sutures while one is waiting to expel the placenta, and tied immediately after its expulsion.

The insertion of the perineal-laceration sutures at this time is not a simple matter, the mucosa being much engorged and the oozing make the raw surfaces hard to outline, as well as reasons for haste that are often present, frequently makes this operation often anything but a success.

(d) *Allowing the patient to lie on the side*, and insisting on a modified Sims's position entirely after the fifth day, and the assumption of the knee-chest position for three minutes night and morning after the seventh day, assists in allowing the uterus to fall forward by gravity in anterversion.

Though patients complain much of the inconvenience at times, through fifteen years of trial I have become to think it more and more important both for the natural involution, both of the uterus, and also of the ligaments, which latter are by no means (as I have pointed out) a negligible quantity.

The remaining in bed *two weeks* for a normal expectant delivery and three to three and one-half weeks for an operative, I believe to be a good plan where possible.

(e) *Episiotomy* should always be thought of as a prophylactic operation to avoid as much as possible the tearing of the perineum; better a bilateral episiotomy than a perineal laceration, though one can not always avoid tears even then. The episiotomy incision is made from the side of the introitus in a radial incision in an imaginary line drawn from the middle of the presenting part toward the muberosity of the ischium better with a pair of sharp scissors than a knife. It is out of the way of the iochia and heals by first intention if sutured and even if not sutured usually heals without a great deal of deformity. Care must always be taken to divide the tense mucosa equally with the muscle (cavernosi) and skin, to attain the required extra space.

(f) *Perineorrhaphy and colporrhaphy* after *six-tenth weeks after labor* is done in many different patterns of denudation Hegar's triangle, Thomas' butterfly, Martin's or the Doleris, or Tait procedure are often of great importance as to choice.

The simple denomination of the mucosa and narrowing of the introitus are of small importance compared to the true principle involved.

To simply denude a part of the mucosa and reunite these edges is much as if one denuded the skin over an inguinal hernia and covered the raw surfaces by tight skin to hold back a reducible hernia.

The true principle is to restore the calibre and unite the fascia and muscle where possible for the support of the ampulla recti.

As perineorrhaphy will not restore the position of the uterus and to simply narrow below will not stop prolapsus, especially if the uterus be retroverted, indeed to shorten the vagina simply may aid the prolapsus unless the ampulla is well supported and even though the floor be perfectly patched, without the ligamentous support necessary it is of little use.

(g) *A pessary* in retroversion (non-adherent) is often of value in holding the cervix posteriorly, so that the corpus may fall forwards, and though recently fallen into disuse, it in certain cases has a real value, both in those not requiring operation as well as those after the pereneal operation.

The introduction should be made in the usual manner with the patient on the back, then the genu-rectoral position should be assumed and the final adjustment then made, when both pessary and uterus will assume a position that it is impossible to attain by adjusting while on the back; the patient, by assuming the knee-chest position daily, can aid materially in maintaining the pessary in a proper position and the uterus also. Frequently several trials are needed before a correctly fitting pessary is adjusted.

The operations for shortening the round ligaments, either externally by the Adams-Alexander operation of internally can be tried. By these two means we are able to attain the result of keeping the uterus anteverted and narrowing and perhaps correcting somewhat the vaginal direction, but the needs of the cases are frequently far from being adequately filled.

(h) The *Adams-Alexander* operation, by shortening the round ligaments should never be done without introducing a pessary to hold the cervix back to allow the body to fall forward, and indeed needs well selected cases by a competent broad ligament histopathologist, to be a success as an operation, and often there are many other factors that this procedure is entirely incapable of combating, therefore placing it in the category of supplementary operations.

(i) *The Wylie, Pryor Ferguson or Clarence Webster intra-abdominal* operations give one a better opportunity for the inspection of the intra-pelvic conditions, as well as a better chance for some modification to suit the needs of the case; indeed there may be other conditions that need to be corrected, anyway it is simply shortening a natural support, and consequently, conservatively scientific, though Ferguson's idea will not appeal as rational if we remember that the sound ligaments allow the rotation of the pregnant uterus as it rises out of the pelvis, and it seems to me that the Ferguson operation might interfere with this taking place naturally, and if so, is certainly contra-indicated during the reproductive period, and is also another plea for the gynecologist and obstetrician to be united in their special work.

*The anterior suspension* or fixation should perhaps not be done in people still capable of becoming pregnant, and certainly not with apparent reckless abandon of the past, and many times the cases where there was no need of it, with the awful sequelæ during pregnancy or parturition; indeed, it becomes us ill at any time to substitute one pathological condition to correct another which this is, by anchoring the uterus whose very office it is to change in size and capacity to which end it is situated in the loose para-metrium, with the loose para-proctium behind and para-cystium in front.

*Hysterectomy* may indeed have to be done, and even then a perineorrhaphy and colporrhaphy with anchoring of the vagina may be needful.

In other old inoperable cases, we are often forced to resort to the simple Scanzoni hysterophore for support.—*Chicago Policlinic, Chicago, Ills., February, 1905.*

